# Melanotic Schwannomas Are Rarely Seen Pigmented Tumors with Unpredictable Prognosis and Challenging Diagnosis

**DOI:** 10.1155/2017/1807879

**Published:** 2017-10-03

**Authors:** Elif Keskin, Sumeyye Ekmekci, Ozgur Oztekin, Gulden Diniz

**Affiliations:** Tepecik Education and Research Hospital, Izmir, Turkey

## Abstract

Melanotic Schwannoma (MS) is rarely seen and potentially malignant neoplasm that is categorized as a variant of Schwannoma. MS most frequently involves intracranial structures followed by posterior nerve roots in the spinal canal. Approximately 50% of the cases with MS have psammomatous calcifications and this type of MS is related to Carney complex with autosomal dominant inheritance. Most cases of MS are benign, though 10% of them are malignant with metastatic potential. MS mimics melanoma and the differential diagnosis should be made excluding other melanin producing neoplasms especially melanoma.* Case 1*. A 42-year-old hypertensive male presented for checkup. He had a well-defined extraspinal oval lesion measuring 3.5 × 2.5 cm near right adrenal.* Case 2*. A 22-year-old female presented with neurofibromatosis-2, bilateral acoustic schwannomas and café au lait lesions on sacrococcygeal region. She had an intradural extramedullary lesion measuring 6.1 × 2.0 cm at L1-2 level. MS is a rare neoplasm composed of Schwann cells and melanin pigment. These tumors are usually benign but they may become aggressive. The biologic behavior of MS is difficult to predict; the patients have to be followed up for a longer period due to its malignant potential.

## 1. Introduction

Melanotic Schwannoma (MS) is a rare, distinctive, potentially malignant neoplasm that is categorized as a variant of Schwannoma [1, 2]. MS was first described by Hudson in 1961 with only 100 cases reported in the literature so far [3]. Most MS cases affect intracranial structures, followed by posterior nerve roots of the spinal canal, and rarely sympathetic chain, acoustic nerve, cerebellum, orbit, choroid, soft tissue, heart, oral cavity, esophageal wall, stomach, bronchus, retroperitoneum, uterine cervix, and parotid gland [3, 4]. Spinal MS arises in the lumbosacral region (47.2%) and thoracic (30.5%) and cervical (22.2%) levels and rarely intramedullary types are seen. Male-female ratio is 1.1 and age of the patients ranges between 10 and 84 years; however its highest frequency is in the fourth decade [3].

Approximately 50% of the cases with MS have psammomatous calcifications with a benign course which is related to Carney complex with autosomal dominant inheritance [1]. Carney complex is associated with lentiginous pigmentation, myxomas, endocrine overactivity, and cutaneous blue nevi [5]. Most cases of MS are benign though 10% of them are malignant with metastasis that is characterized by epithelioid cells with variably sized nuclei and marked accumulation of melanin in neoplastic cells and melanophages [2, 5]. MS mimics melanoma and the main differential diagnosis is made excluding other melanin producing neoplasms especially melanoma [2].

Herein, we are presenting two cases of MS originating in the retroperitoneal (paraspinal) and spinal canal and discussing its possible clinical and pathologic associations in the light of the present literature.

## 2. Case Report

**Case 1 .**

A 42-year-old hypertensive male presented for checkup. Neurological and physical examination as well as family history were noncontributory. Abdominal computed tomography revealed a well-defined extraspinal oval lesion with amorphous calcifications near right adrenal measuring 3.5 × 2.5 cm and compressing inferior vena cava. Surgical excision was performed.

**Figure 1:**
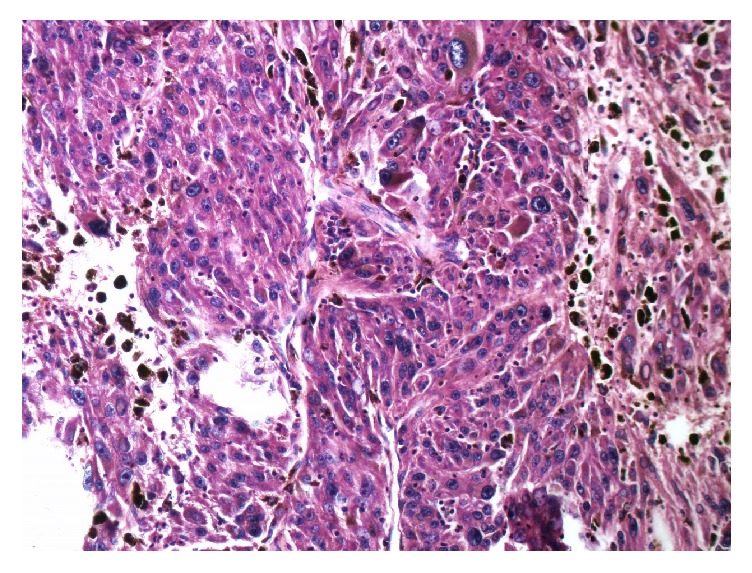
Pigmented pleomorphic tumor cells in Case 1 (HEX 200).

**Figure 2:**
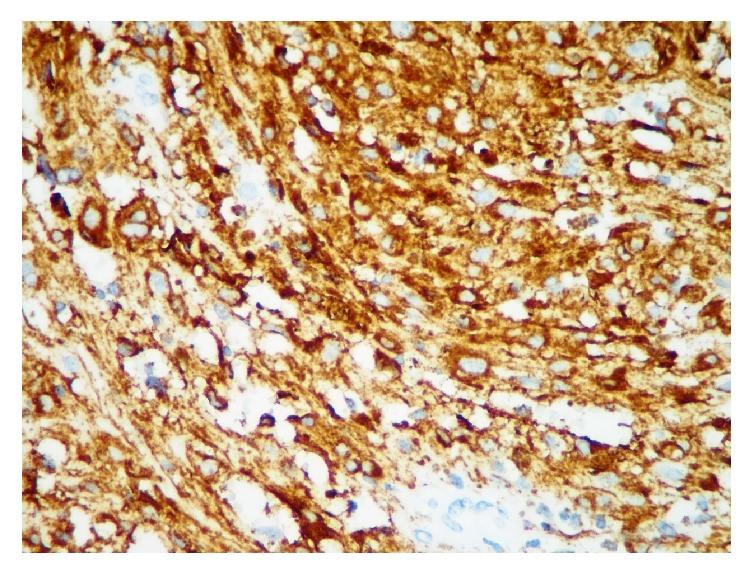
Presence of diffuse HMB45 expression (DABX 200).

Gross examination disclosed encapsulated, brown-black solid tumor measuring 3.8 × 2.8 × 2.4 cm with a smooth surface. On histological examination pleomorphic tumor cells were arranged in syncytial pattern (Figure 1). Tumor cells had intranuclear cytoplasmic pseudoinclusions and variable amounts of cytoplasmic melanin pigments. A few mitoses were observed up to 1/10 high power fields. Immunohistochemical staining for human melanoma black 45 (HMB45), Melan A, Vimentin, S-100 protein, Cytokeratin, CD56, Chromogranin, Synaptophysin, and Ki 67 was performed. Tumor cells showed diffuse and strong expression of HMB45 (Figure 2), Melan A, and S-100 protein but were negative for Cytokeratin, CD56, Chromogranin, Synaptophysin. Ki 67 was positive in 5% of the tumor cells.

**Case 2 .**

**Figure 3:**
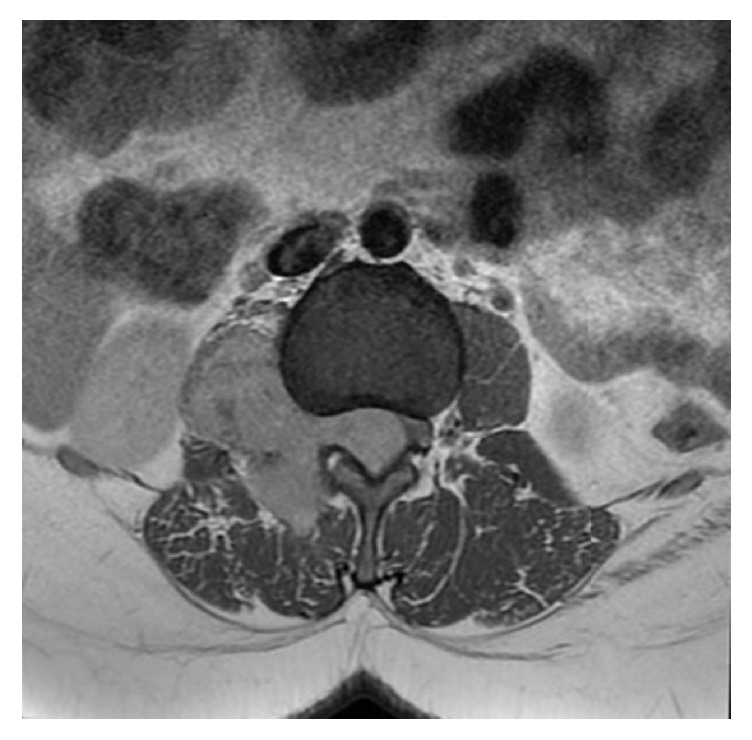
In T1-contrasted axial series, extraspinal extension of the tumor from the spinal canal through the neural foramen is seen.

**Figure 4:**
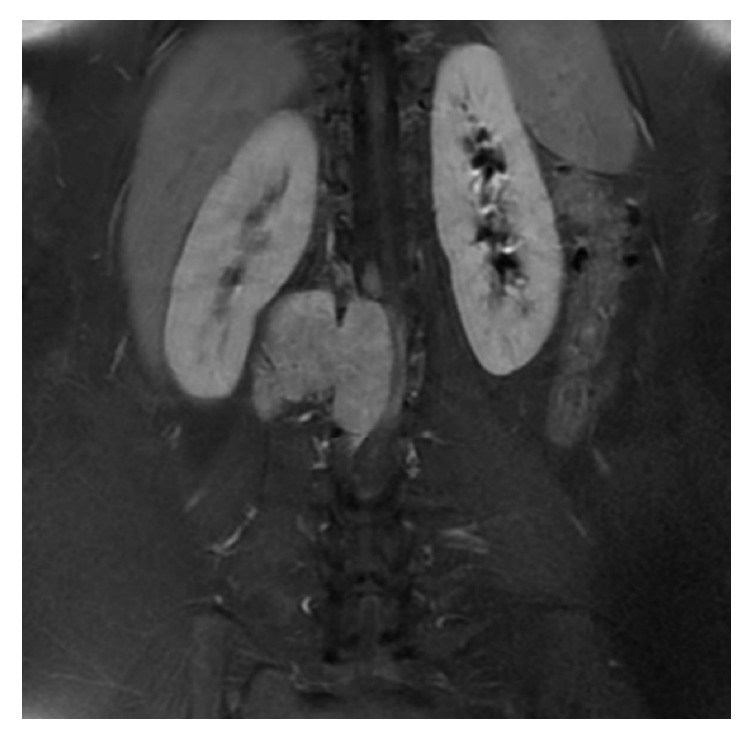
In T1-contrasted coronal series, dumbbell shape extraspinal extension of tumor is monitored.

**Figure 5:**
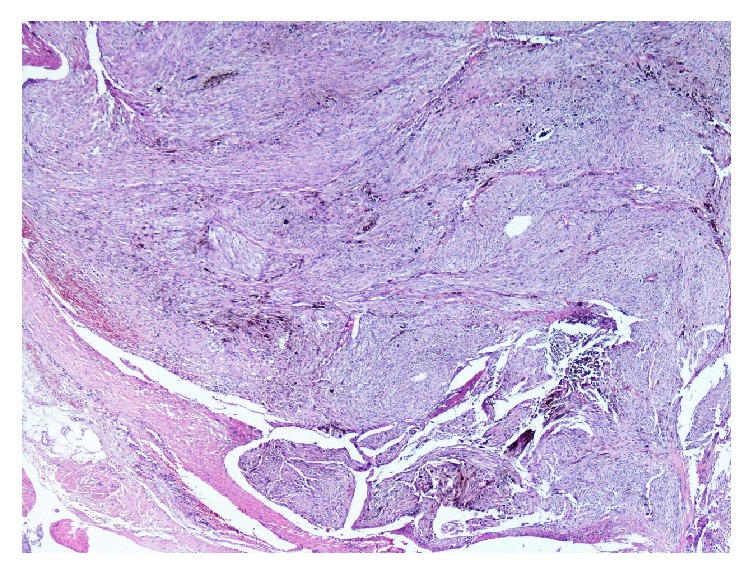
Panoramic appearance of tumor with infiltrative border (HEX 20).

**Figure 6:**
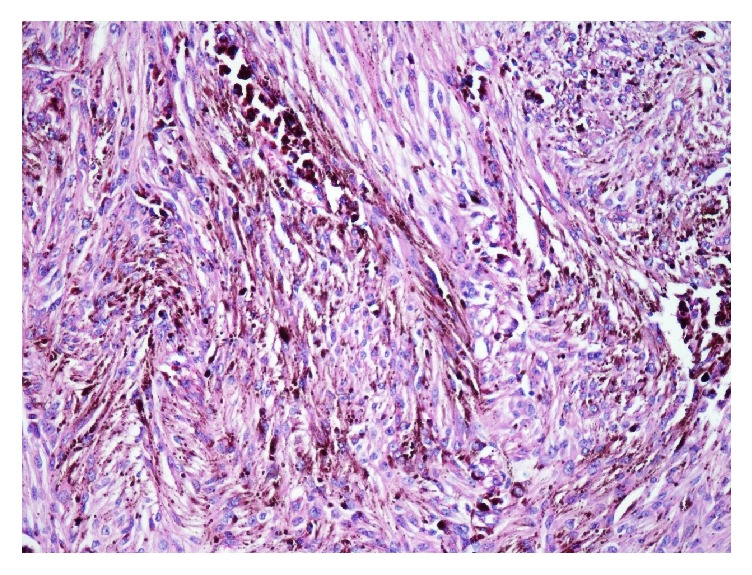
Tumor of Case 2 is composed of mostly spindle cells (HEX 200).

**Figure 7:**
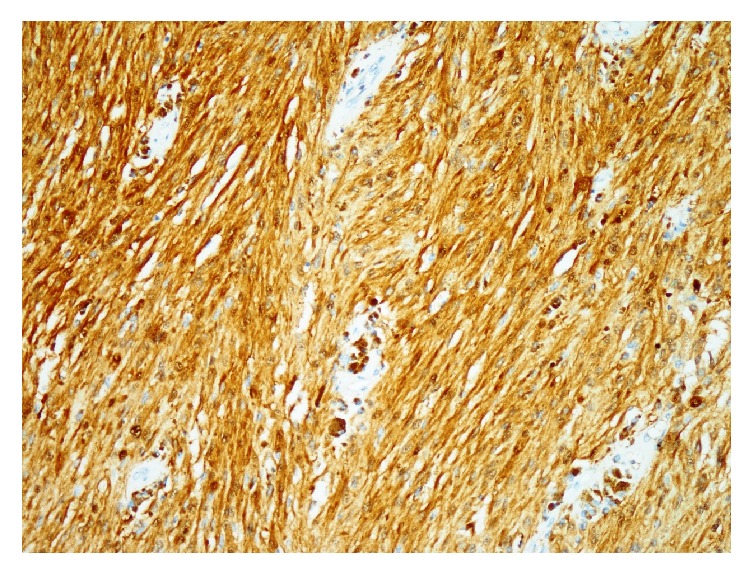
Presence of diffuse S-100 protein expression (DABX 200).

A 22-year-old female presented with lumbar pain. She had neurofibromatosis-2, bilateral acoustic Schwannomas, and café au lait lesions on the sacrococcygeal region. She was operated on with the indication of intraconal orbital meningioma. Magnetic resonance imaging (MRI) of lumbar spine revealed intradural extramedullary lesion measuring 6.1 × 2.0 cm at L1-2 level near the right kidney which was hypointense on T1- and hyperintense on T2-weighted sequences (Figures 3 and 4). Surgical excision was conducted to visualize the lesion. Histopathology revealed spindle cell tumor arranged in lobular pattern with infiltrative border (Figure 5). Tumor cells had distinctive nucleoli and melanin pigments in cytoplasm (Figure 6). Mitoses were seen on 1/10 high power fields. Immunohistochemical staining for HMB45, Melan A, Vimentin, S-100 protein (Figure 7), Cytokeratin, EMA, Actin, Desmin, and Ki 67 was performed. Tumor cells showed diffuse and strong expression of HMB45, Melan A, Vimentin, and S-100 protein but were negative for Cytokeratin, EMA, Actin, and Desmin. Ki 67 was positive in 1-2% of the tumor cells.

## 3. Discussion

Melanotic Schwannoma can be divided into psammomatous and nonpsammomatous types and about half of the cases with psammomatous MS are related to Carney complex [1]. While nonpsammomatous MS is considered to be a sporadic type, encountered in fourth decades, psammomatous melanotic Schwannoma tends to occur at an earlier age (average age, 22.5 years). Psammomatous type is associated with Carney Syndrome that is characterized by the presence of myxomas in the heart, breast, uterus, skin lentigines, endocrinopathy (Cushing's syndrome), growth hormone producing pituitary adenoma, epithelioid blue nevi, Sertoli cell tumors of the testis, tumors of the thyroid, and ductal adenomas of the breast [1, 4, 6]. MS does not necessarily demonstrate psammoma bodies. Approximately 10% of melanotic Schwannomas have no psammoma bodies and may display a malignant change [7]. In the present study malignant changes or metastases were not detected.

MS is a variant of Schwannoma. It is a circumscribed or encapsulated, black-brown, blue, or gray-colored tumor on gross examination [5]. Microscopic characteristics of this tumor consist of spindle or epithelioid cells with pigmented granules and rare mitotic figures [5, 8]. Its melanin pigment varies from area to area. MS is differentiated from classic Schwannoma with an absence of a distinct capsule and clear-cut Antoni A and Antoni B areas. Also latter one lacks melanin and psammoma bodies [7]. Theories about pathogenesis of MS which try to explain melanin producing properties of the cells include phagocytosis of melanin by Schwann cells, neoplastic differentiation of neural crest cells into Schwann cells with melanogenetic properties, and melanocytic transformation of previously normal Schwann cells [3, 9].

The differential diagnosis of a melanotic Schwannoma is made excluding malignant melanoma, pigmented meningioma and neurofibroma, rhabdomyosarcoma, clear-cell sarcoma of soft tissue, melanotic medulloblastoma, ganglioneuroblastoma, ectomesenchymoma (triton tumor), neurotrophic melanoma, and melanotic neuroendocrine carcinomas and carcinoids [5]. The main problem in the differential diagnosis is to distinguish MS from a metastatic malign melanoma. Though in the present cases immunophenotypic characteristics were consistent with the diagnosis of melanoma, the morphologic feature did not support this diagnosis, and the extremely low index of Ki 67 did not suggest the presence of melanoma. In addition, histologic features of ample cytoplasm, indiscernible cell border, and low proliferative index contribute to the diagnosis of MS rather than malignant melanoma [1]. The melanin pigment makes it difficult to distinguish among cellular details and Ki 67 might be overestimated. In the present cases the tumors had histologic atypia (nuclear pleomorphism, prominent macronucleoli) without necrosis. The mitotic count was up to 1/10 HPFs.

MS derived from the spinal nerve root is characterized by slow growth and low aggressiveness and assumes a dumbbell appearance [6]. Conventional Schwannomas are hypointense on T1-weighted and hyperintense on T2-weighted sequences, contrary to melanotic Schwannomas which are hyperintense on T1-weighted and hypointense on T2-weighted sequences [3, 4]. Our second case was hypointense on T1-weighted and hyperintense on T2-weighted sequences.

Optimal surgical approach is complete tumor resection which is not possible all the time, owing to the local infiltration and the need for adjuvant postoperative radiotherapy and chemotherapy to prevent local recurrences and metastases [4, 5]. Our first case did not demonstrate recurrences or metastases for 30 months.

The natural history of this sort of lesion is uncertain. Advanced age seems to predict poor prognosis in sporadic forms [3]. Presence of mitosis over 1/10 HPFs is the other known risk factor for metastatic MS [1]. Recently some researchers have suggested that the malignant potential of melanotic Schwannomas (with or without psammoma bodies) is underestimated [10]. The biologic behavior of MS is difficult to predict, and the patients have to be followed up for a longer period due to its malignant potential [8].

## 4. Conclusion

MS is a rare neoplasm composed of Schwann cells and melanin pigment. These tumors are usually benign but they may become aggressive and metastatic. Total excision and long-term follow-up are recommended including screening for Carney's syndrome especially in young patients.
